# Mesopolysaccharides: The extracellular surface layer of visceral organs

**DOI:** 10.1371/journal.pone.0238798

**Published:** 2020-09-17

**Authors:** Willi L. Wagner, Yifan Zheng, Aidan Pierce, Maximilian Ackermann, Heinz Horstmann, Thomas Kuner, Paolo Ronchi, Yannick Schwab, Philip Konietzke, Felix Wünnemann, Mark O. Wielpütz, Hans-Ulrich Kauczor, Steven J. Mentzer

**Affiliations:** 1 Department of Diagnostic and Interventional Radiology, University of Heidelberg, Heidelberg, Germany; 2 Translational Lung Research Center, Member of the German Center for Lung Research, University of Heidelberg, Heidelberg, Germany; 3 Laboratory of Adaptive and Regenerative Biology, Brigham & Women’s Hospital, Harvard Medical School, Boston MA, United States of America; 4 Institute of Functional and Clinical Anatomy, University Medical Center of the Johannes Gutenberg-University, Mainz, Germany; 5 Department of Functional Neuroanatomy, Institute for Anatomy and Cell Biology, University of Heidelberg, Germany; 6 European Molecular Biology Laboratory, Electron Microscopy Core Facility, Heidelberg, Germany; 7 European Molecular Biology Laboratory, Cell Biology and Biophysics Unit, Heidelberg, Germany; VIT University, INDIA

## Abstract

The mesothelium is a dynamic and specialized tissue layer that covers the somatic cavities (pleural, peritoneal, and pericardial) as well as the surface of the visceral organs such as the lung, heart, liver, bowel and tunica vaginalis testis. The potential therapeutic manipulation of visceral organs has been complicated by the carbohydrate surface layer—here, called the mesopolysaccharide (MPS)—that coats the outer layer of the mesothelium. The traditional understanding of MPS structure has relied upon fixation techniques known to degrade carbohydrates. The recent development of carbohydrate-preserving fixation for high resolution imaging techniques has provided an opportunity to re-examine the structure of both the MPS and the visceral mesothelium. In this report, we used high pressure freezing (HPF) as well as serial section transmission electron microscopy to redefine the structure of the MPS expressed on the murine lung, heart and liver surface. Tissue preserved by HPF and examined by transmission electron microscopy demonstrated a pleural MPS layer 13.01±1.1 um deep—a 100-fold increase in depth compared to previously reported data obtained with conventional fixation techniques. At the base of the MPS were microvilli 1.1±0.35 um long and 42±5 nm in diameter. Morphological evidence suggested that the MPS was anchored to the mesothelium by microvilli. In addition, membrane pits 97±17 nm in diameter were observed in the apical mesothelial membrane. The spatial proximity and surface density (29±4.5%) of the pits suggested an active process linked to the structural maintenance of the MPS. The striking magnitude and complex structure of the MPS indicates that it is an important consideration in studies of the visceral mesothelium.

## Introduction

The mesothelium is a dynamic and specialized tissue layer that covers 3 somatic cavities (pleural, peritoneal, and pericardial) and the surface of the visceral organs such as the lung, heart, liver, bowel and tunica vaginalis testis. In developing tissues, the mesothelium gives rise to mesenchymal cells in a critical process of mesothelial-mesenchymal transition (MMT) [1, 2]. In adulthood, the mesothelium regulates physiological responses to disease, infection and injury [3]. Mesothelial cells participate in innate immune responses (e.g. via processes mediated by toll-like receptors) [4, 5], secrete relevant inflammatory mediators [6, 7], present antigen to immune cells [6, 7], control plasminogen activation [8], regulate extracellular matrix production [9], secrete growth factors [10] and transform the organ surface through adult MMT [1].

The potential for therapeutic manipulation of these diverse functions has been complicated by the presence of a carbohydrate surface layer—here, called the mesopolysaccharide (MPS)—that coats the outer layer of the mesothelium. Although the structure and function of the MPS is not fully-understood, a common understanding is that the MPS creates a frictionless surface facilitating the movement of the ventilating lung, beating heart and peristaltic bowel [11, 12].

Our current understanding of MPS structure has been largely based on conventional fixation techniques used for transmission electron microscopy (TEM) sample preparation. Using conventional chemical fixation and ruthenium red staining, Andrews and Porter described a 150 Å thick surface coat on mesothelial cell surfaces and between microvilli [13]. Wang used cationic colloidal iron staining to infer the presence of negatively charged sialomucins on the mesothelial surface [14, 15]. Ohtsuka et al. demonstrated that digestion of the surface carbohydrates with neuraminidase or hydrolysis of the sialic acid with H_2_SO_4_ prevented colloidal iron staining [16]. These observations indicated that the MPS is characterized by strong anionic sites; however, the destructive effects of traditional fixation techniques have limited our understanding of the magnitude and structure of the MPS.

Fixation methods, such as vitrification by high pressure freezing followed by freeze substitution [17], provide an opportunity to re-examine the structure of both the MPS and the visceral mesothelium. In this report, we used high pressure freezing (HPF) as well as conventional fixation methods in combination with ruthenium red staining to redefine the morphological complexity of the mesothelial and MPS surface layer of the murine lung, heart and liver.

## Results

**Fig 1:**
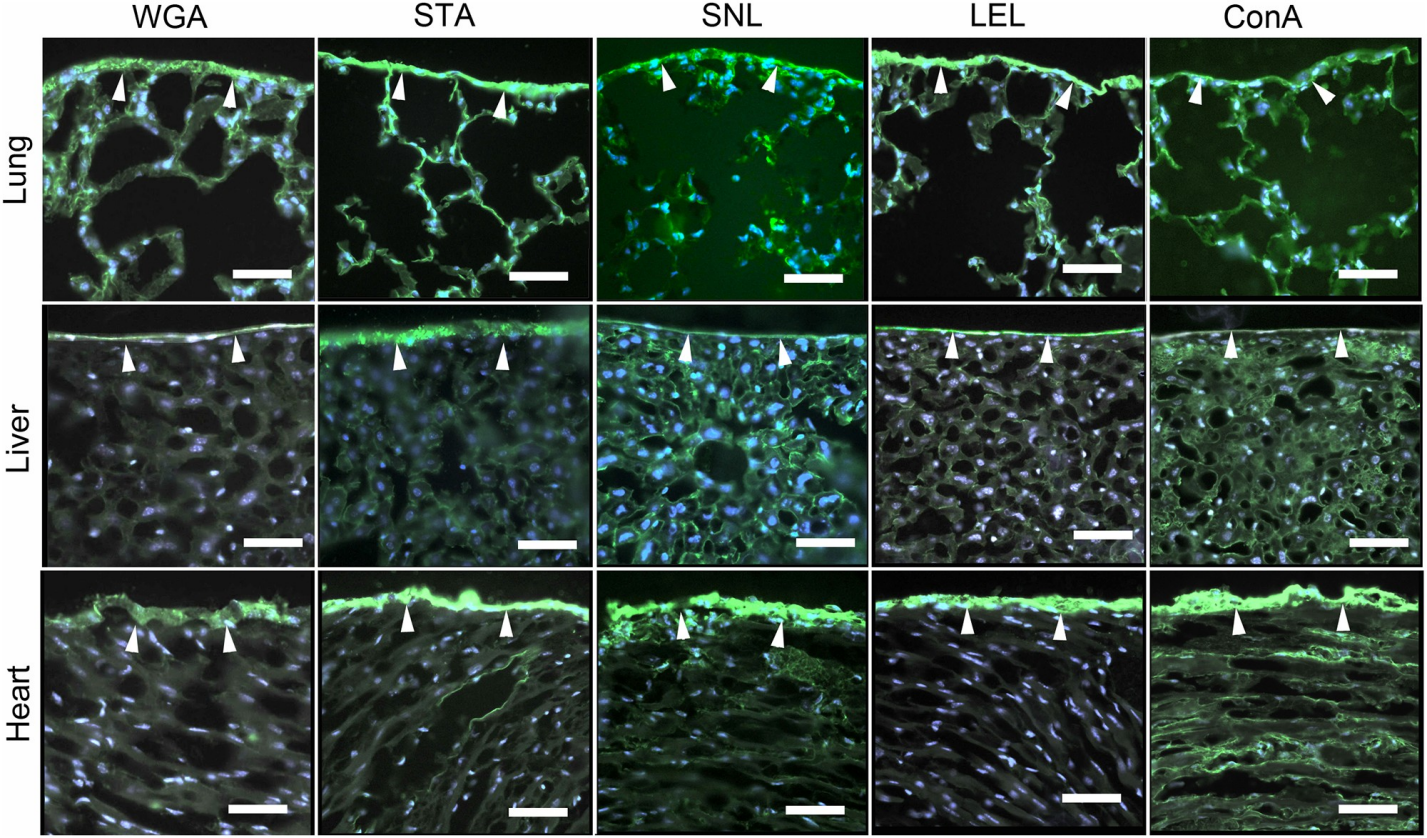
Fluorescent lectin staining of the mesopolysaccharides of the lung, heart and liver. Frozen tissue sections were warmed to 27°C before fixation in 2% paraformaldehyde. The lectin fluorescence detection was performed as described in Materials and Methods and images were obtained with fluorescent microscopy. The lectin staining was demonstrated with fluorescently labeled Concavalia ensiformis (ConA) [18], Lycopersicon esculentum lectin (LEL) [19], Sambucus nigra (SNL) [20], Solanum tuberosum (STA) [21] and wheat germ agglutinin (WGA) [22].

The carbohydrate layer expressed on the surface of the murine lung, heart and liver was revealed by a variety of different fluorescent lectins. The lectin panel demonstrated prominent staining of all three organs (Fig 1).

**Fig 2:**
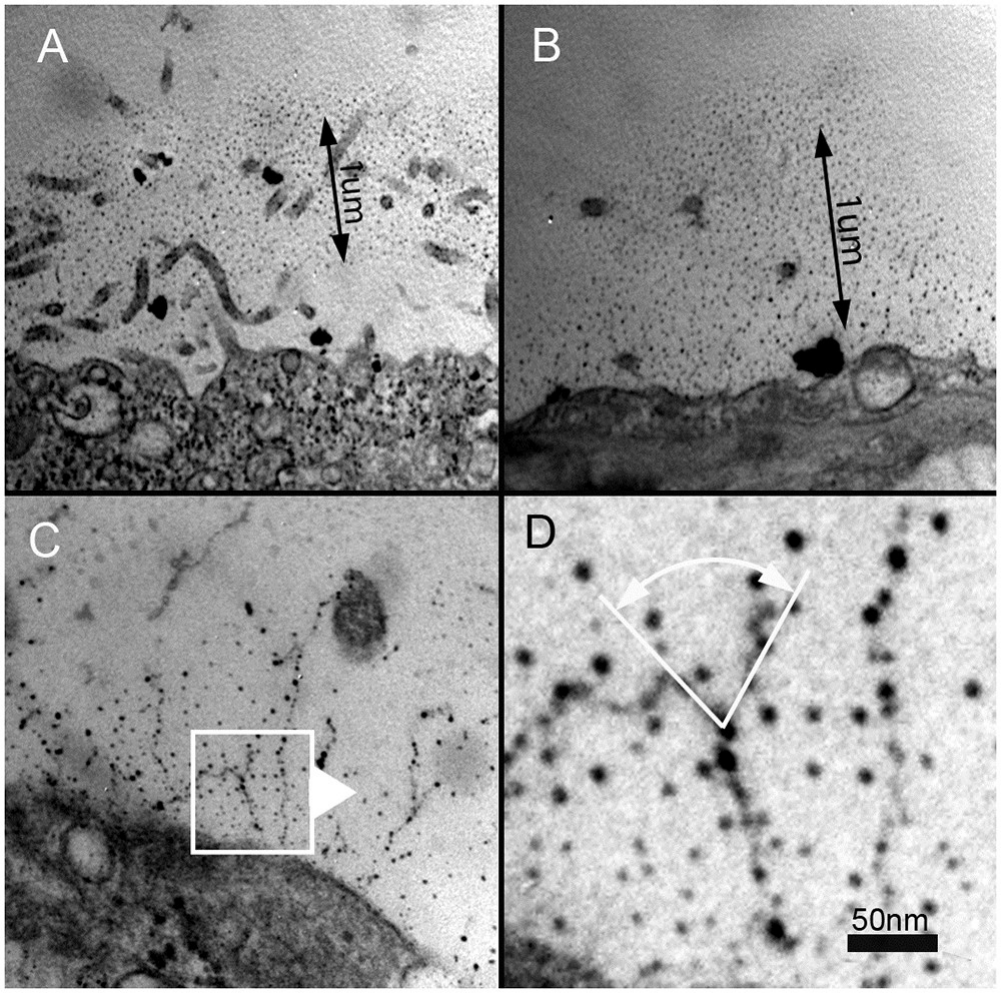
Chemical fixation, ruthenium red staining and transmission electron microscopy of the murine visceral pleura. A,B) The ruthenium-stained carbohydrate layer appears to extend approximately 1um from the pleural mesothelial surface. C,D) Detailed analysis of the images suggested that the MPS is composed of beaded strands with a branching structure.

To study the structure on the visceral pleura of the lung by TEM, we initially used the traditional carbohydrate stain ruthenium red [23]. The TEM images demonstrated an indistinct MPS layer with individual MPS strands measuring 0.34±0.13 μm in length and 21.65±5.39 nm in diameter. The MPS structure demonstrated a backbone with a branching pattern, increased density near mesothelial microvilli and a beaded periodicity (MPS bodies) (Fig 2). The ruthenium red staining of the MPS was independent of the use of osmium tetroxide (OsO_4_) as a tissue fixative. When compared with lectin staining, conventional staining with ruthenium red and thorium dioxide appeared to underestimate the scale of the MPS.

**Fig 3:**
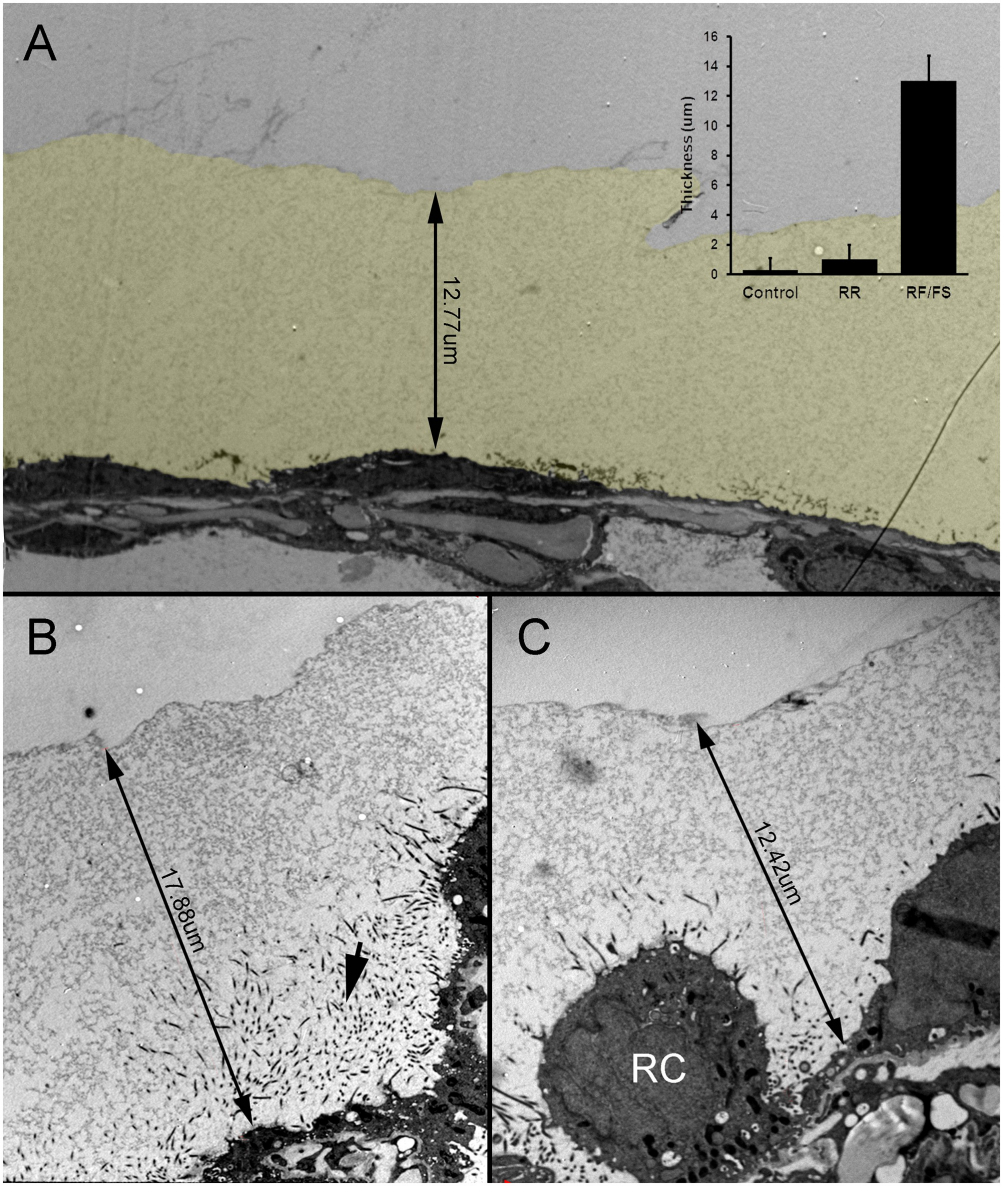
HPF preservation of the MPS expressed on the murine visceral pleura. A) The MPS was several-fold thicker than the underlying mesothelium and significantly thicker than the glycocalyx detected by conventional ruthenium red (RR) staining (control) (inset p<.0001). The MPS is pseudocolored yellow for presentation purposes. B-C) HPF fixation of the visceral pleura demonstrated MPS thickness ranging from 8um to 18um. Microvilli, seen in cross-section, extended into the MPS from the associated mesothelium (B, arrow). Notably, the MPS was present even in the setting of activated or reactive (RC) underlying mesothelial cells [25].

To more accurately characterize the structure of the MPS, we adapted high pressure freezing (HPF), a cryogenic fixation technique that preserves tissue ultrastructure [17]. HPF mitigates the disruptive effects of chemical fixation to more accurately define the structure of the MPS. HPF is a technique that uses liquid nitrogen under high pressure (2100 bar) to rapidly freeze the specimen and preserve ultrastructural detail by avoiding ice crystal formation—a process known as vitrification [24]. Freeze substitution was used to fix the samples at low temperature and provide resin embedded blocks which can be processed at room temperature. This preservation protocol showed a striking difference in the preservation of MPS both in appearance and abundance. As HPF allows MPS to be preserved in its hydrated state before fixation and resin embedding, the appearance can be considered closer to the native appearance of MPS. Representative MPS layers were 13.0±1.1um thick—a 100-fold increase in depth compared to what could be observed using conventional chemical fixation techniques [13] (Fig 3).

**Fig 4:**
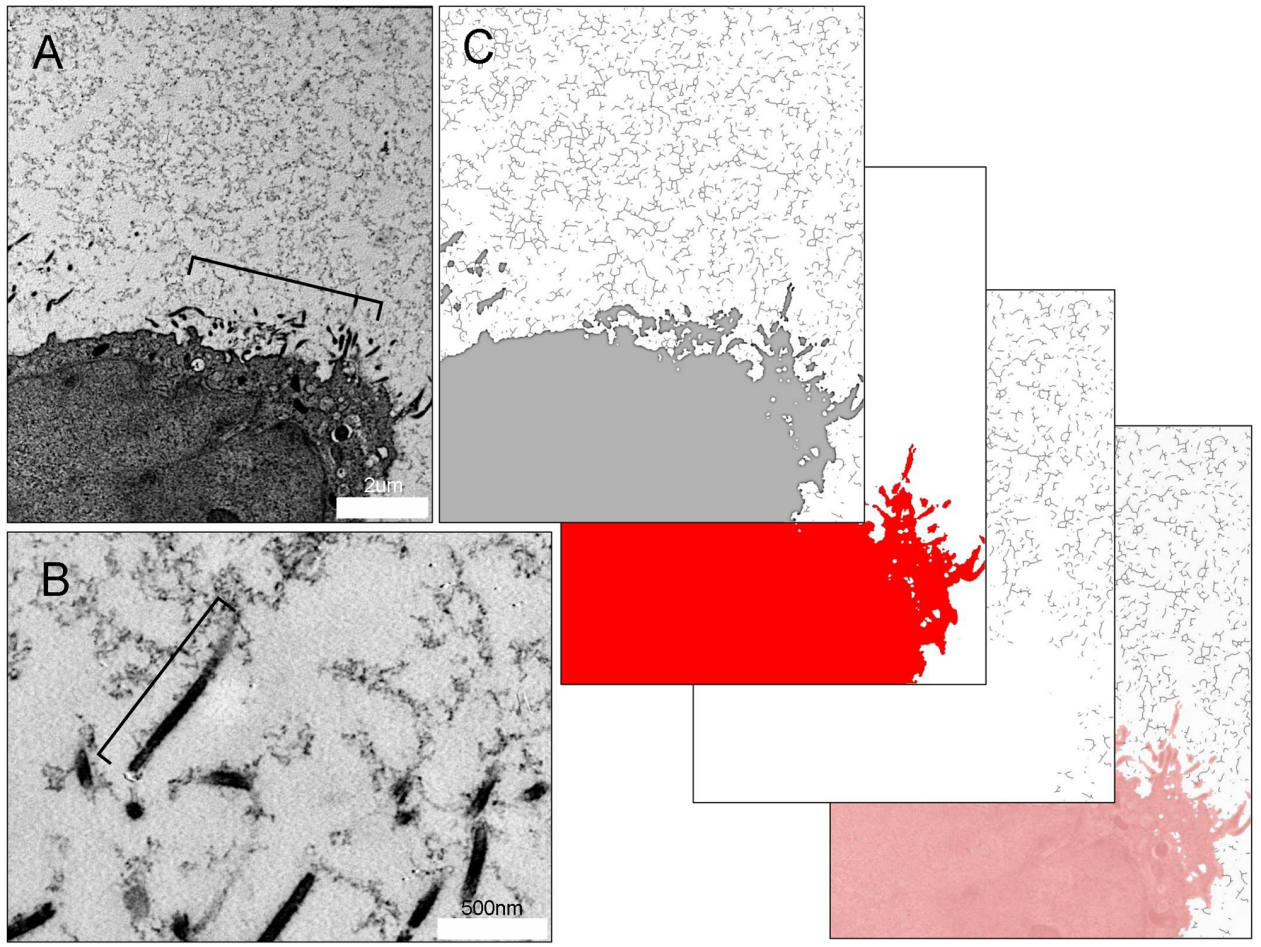
Mesothelial microvilli anchoring the MPS of the visceral pleura. A) HPF TEM of mesothelial cell microvilli (bracket) and surrounding MPS in tissues preserved by HPF. B) Magnification of the microvilli demonstrates traces of the MPS linked to the microvilli (bracket). Cross-section of the pleura imaged with TEM. The microvilli extend 1.1 ± 0.35 um from the mesothelial surface. C) Image analysis using a skeletonization algorithm illustrates the branched-chain structure and connectivity of the carbohydrate biopolymer with an average branch number of 2.77 branches, a mean branch length of 134.86 ± 81.70 nm and a Euclidean distance of 115.65 ± 65.46 nm between skeletonized branches.

Morphological evidence suggested that the MPS was anchored to the visceral pleura by microvilli (Fig 4A, bracket). The pleural microvilli were 1.1±0.35 um long and 42±5 nm in diameter. Microvillar density was 623 microvilli per 10^2^ um of the mesothelial surface. HPF preserved tissues also revealed multiple sites of MPS attachment to the microvilli (Fig 4B, bracket). Image analysis of the MPS using a skeletonization algorithm demonstrated a branched-chain structure characteristic of a unique class of polysaccharides [26]. The branched-chain MPS demonstrated extensive connectivity with a mean branch length of 134.86 ± 81.70 nm and a Euclidean distance of 115.65 ± 65.46 nm between skeletonized branches (Fig 4C).

**Fig 5:**
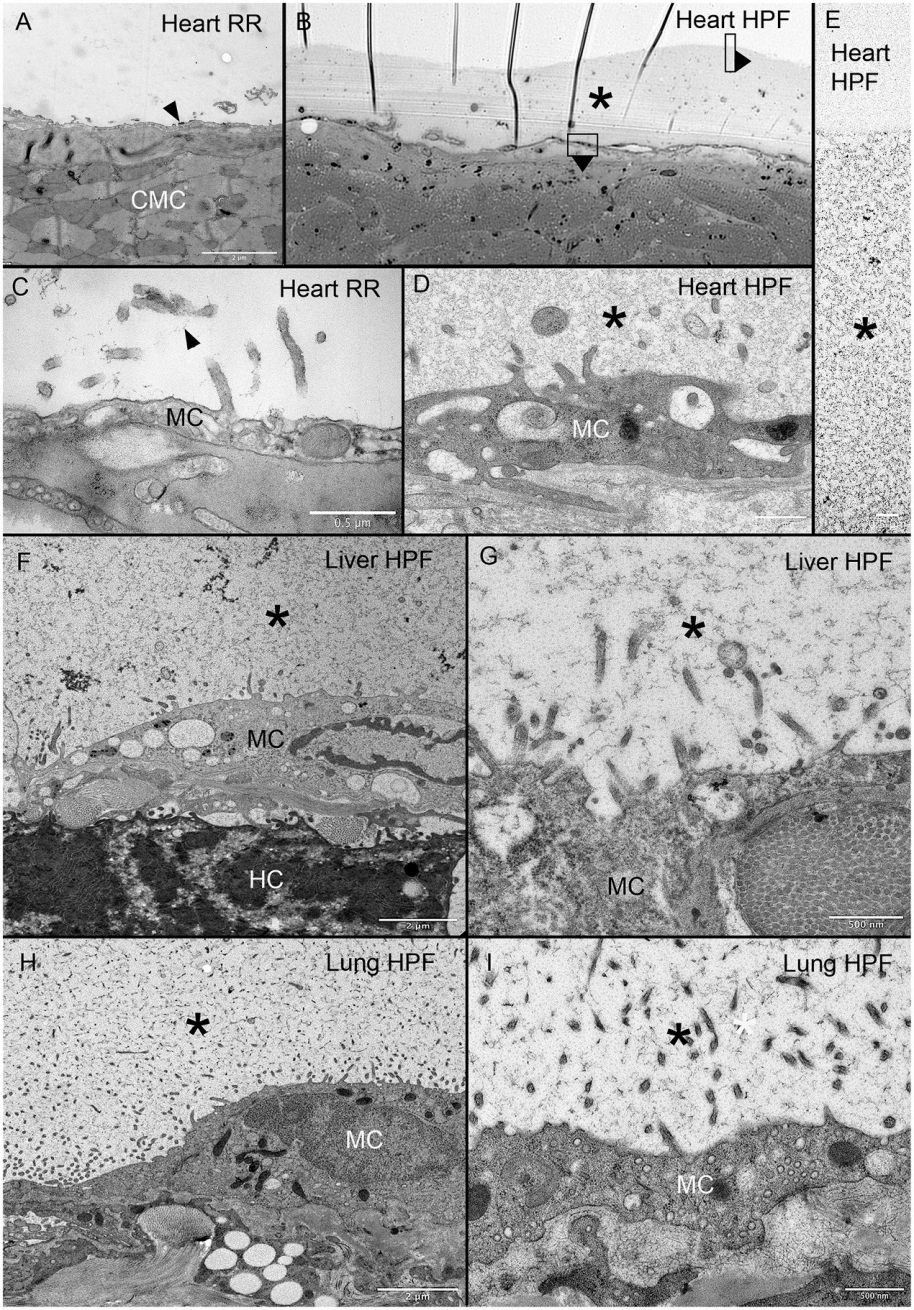
MPS expressed on heart, liver and lung organ surface. Ruthenium red staining resulted in scant contrast detected on the heart surface (A, C arrows). In a light microscopic overview of a 299 nm thick section stained with toluidine blue, the murine heart surface demonstrated a much thicker surface layer (B, asterisk). TEM images of similar areas of the MPS is shown (D, E asterisk). Surface features of the MPS were also similar on the liver (F, G asterisk) and lung (H, I asterisk). Note the similar branched-chain structure in all three organs. RR = ruthenium red; HPF = high-pressure fixation; MC = mesothelial cell; CMC = cardiac myocyte; HC = hepatocyte. Asterisk identifies the MPS in all tissues.

To determine if the MPS was similarly expressed on other visceral organs, we compared ruthenium red staining and HPS on the heart, liver and lung. Consistent with previous observations, the ruthenium red staining on the heart and liver was limited. In contrast, the MPS demonstrated with HPF was consistently greater than four-fold thicker than the underlying mesothelium (Fig 5A–5E). In addition, the HPF technique demonstrated a comparable structural appearance of the MPS in all three organs (Fig 5).

**Fig 6:**
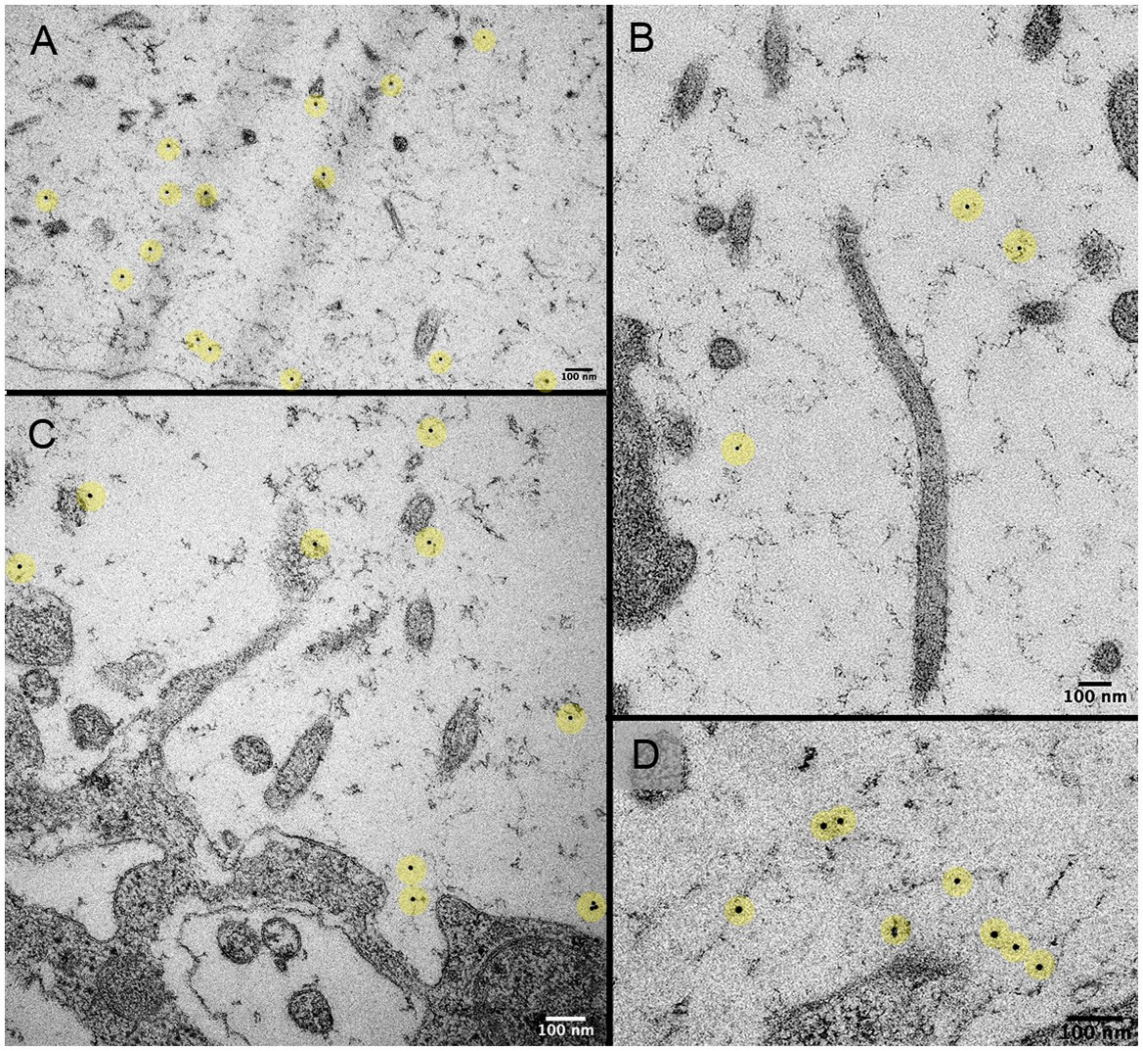
WGA lectin conjugated to 10 nm nanogold binds to the murine pleural MPS. TEM imaging of the nanogold-labeled WGA throughout the MPS (A-D). The nanogold particles are highlighted in yellow. Note, the nanogold-conjugated WGA bound to the MPS and not the microvilli or mesothelial cell surface.

To confirm that the lectin fluorescence originally observed on the organ surface reflected lectin binding to the MPS, we used lectin-nanogold labeling and TEM imaging of the pleural surface. Nanogold-labeled WGA lectin demonstrated prominent binding throughout the MPS (Fig 6). In contrast, nanogold without WGA demonstrated essentially no binding.

**Fig 7:**
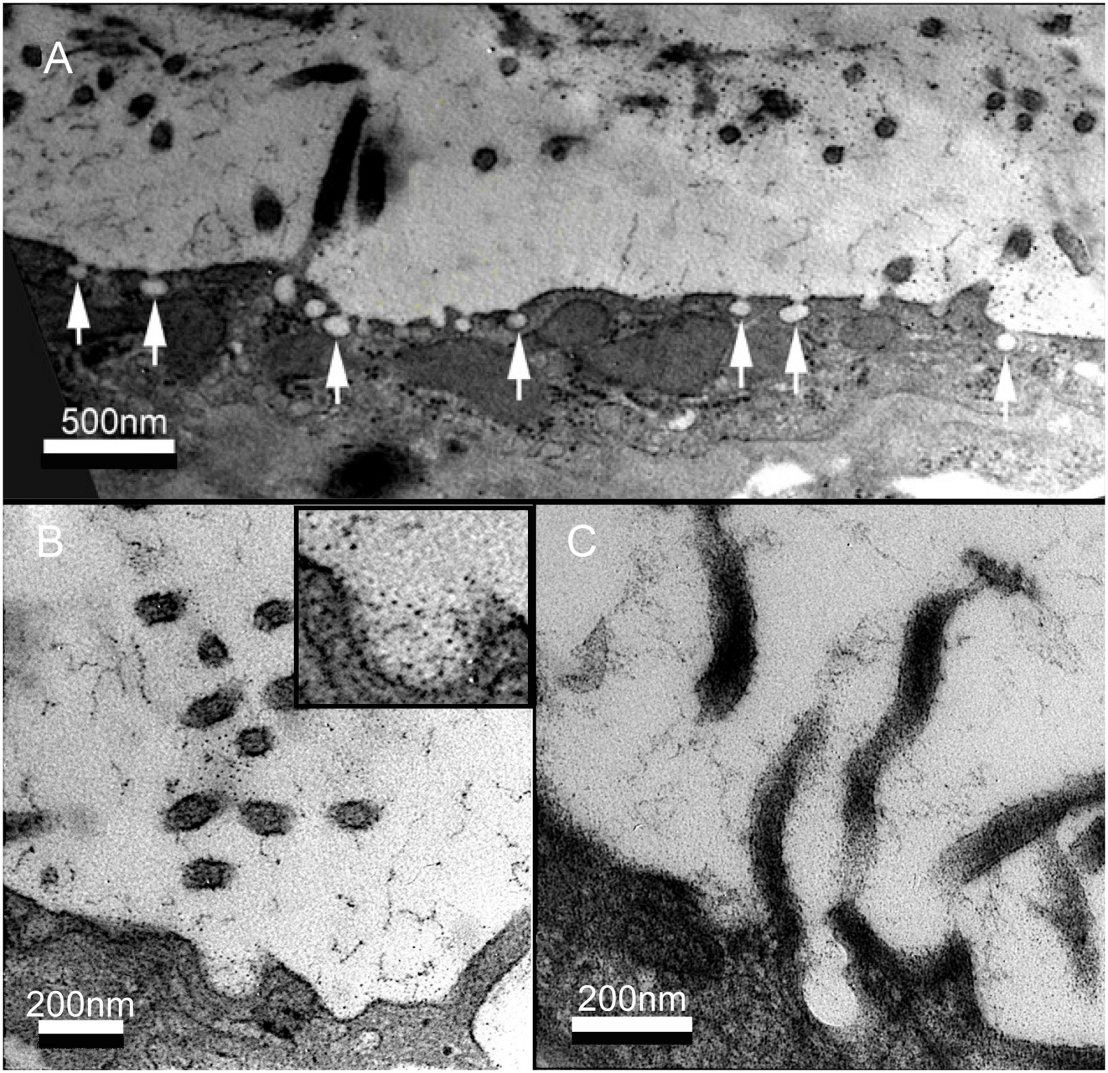
Mesothelial pits and the MPS. A) RR TEM demonstrating pits, 97 ± 17 nm in diameter, on the luminal surface of the mesothelial cells. B, C) Both high magnification RR and HPF TEM demonstrated a close spatial and structural association of the mesothelial pits with microvilli and the MPS.

In addition to characterizing the microvilli and MPS, TEM demonstrated membrane pits at the surface of the mesothelium (Fig 7). The pits had a diameter of 82 ± 7.8 nm with a membrane surface density of 29 ± 4.5%. The pits demonstrated an intimate spatial association with the microvilli and the MPS.

## Discussion

In this report, we used advanced preservation and imaging techniques to demonstrate a previously under-appreciated component of the mesothelial cell surface layer—namely, the mesopolysaccharide (MPS). A conceptual subset of the glycocalyx, the MPS demonstrates several unique features: 1) an underlying branched-chain structure with beaded periodicity (MPS bodies), 2) an intimate structural association with mesothelial microvilli and membrane pits, and 3) a structural scale 100-fold greater than previously appreciated. The striking magnitude and complex structure of the MPS suggests that it is an important consideration in studies of the visceral mesothelium.

A long-standing observation has been the presence of an extracellular polysaccharide-rich coating on both eukaryotic [27] and prokaryotic [28] cells. In 1963, Bennett coined the word glycocalyx (Greek, “sweet husk”) to describe this polysaccharide-rich coating on cells [29]. In mammals, the most studied glycocalyx is expressed on the luminal surface of endothelial cells [30]. Because of the limitations of traditional fixation techniques, studies of the glycocalyx have necessarily focused on the durable portions of the glycocalyx; namely, transmembrane proteoglycans and glycoproteins [31, 32]. Here, we focused on the glycocalyx or carbohydrate surface layer associated with the mesothelium. Because of its unique structure and functional role, we refer to the carbohydrate layer as the mesopolysaccharide or MPS.

To facilitate imaging of the MPS in its natural state, we used a technique of high-pressure freezing (HPF) [33]. This technique of rapid freezing with liquid nitrogen at 2100 bar facilitates vitreous freezing and minimizes the formation of crystalline ice. Subsequent freeze substitution allows for both fixation and preservation of the membrane carbohydrates [34]. In our studies, HPF preserved samples demonstrated an extraordinary thickness of the MPS—more than 100-fold thicker than previous estimates of mesothelial glycocalyx thickness of tissues fixed by conventional chemical methods [13]. A mean thickness of 13 um has implications for both the diffusion of signaling molecules and the accessibility of the mesothelium to cell-cell interactions. For example, structural studies of major histocompatibility complex (MHC) class II antigens suggest that these membrane molecules barely project into MPS (less than 1%) [35]. These observations suggest that the MPS is not only porous to signaling molecules but may play a functional role in cell interactions with the mesothelium.

Both chemically fixed and HPF preserved tissues a high density of microvilli on the mesothelial surface. The microvilli are interconnected by strands of the MPS. The existence of microvilli was first confirmed by electron microscopy in 1954 [36]. Previous studies have demonstrated a wide range of microvillar densities [13, 37–39]. Microvillar density decreases with mesothelial activation [1] and fixation techniques. It is possible that the relatively high density of microvilli observed in this study reflects both the unactivated mesothelium and the good preservation achieved with HPF. Notably, the length and diameter of the microvilli in our study were comparable to previous observations [40–42]. The function of microvilli has been the subject of considerable speculation. The microvilli may function to increase the membrane surface area to facilitate metabolic exchange [36, 43], trap substances in the intervillar regions [44], protect the mesothelial surface from abrasive damage [13] and/or alter the surface membrane charge [36]. In our TEM studies, the numerous polysaccharide attachments underscored at least one role of the microvilli in anchoring the MPS.

An intriguing observation was the bulbous invaginations or pits observed in the apical plasma membrane of the pleural mesothelial cells. Seen in both chemically fixed and HPF preserved tissue, the pits were less than 100 nm in diameter and were interspersed among the surface microvilli. These invaginated membrane structures were similar to, albeit somewhat smaller than, the "vesiculated pits" described by Madison et al. [37] and the "spherical invaginations" described by Baradi and Rao [38] in electron microscopy imaging of peritoneal cell membranes. Notably, these pits are intracellular and not the intercellular stomata of von Recklingshausen [45]. The density of the pits seen on the TEM images suggests a dynamic membrane process; however, it is unclear whether these structures represent endocytic and/or exocytic trafficking at the plasma membrane. Regardless, the occasional staining of carbohydrate contents within the pits suggests that these structures contribute to the maintenance of the MPS.

Finally, there is a relevant structural distinction between the branched-chain structure of the MPS and the linear chains of mucopolysaccharides (glycosaminoglycans) such as hyaluronic acid [46]. We anticipate that our findings will provide a basis for creative chemical techniques to elucidate the composition of the MPS [47, 48]. We speculate that the branched-chain configuration of the MPS, analogous to other structural heteropolysaccharides in biology [49], will be an important consideration in future studies of mesothelial function.

## Methods

### Animals

Male mice, eight to ten week-old wild type C57BL/6 (Jackson Laboratory, Bar Harbor, ME, USA) were anesthetized prior to euthanasia (N = 30). The care of the animals was consistent with guidelines of the American Association for Accreditation of Laboratory Animal Care (Bethesda, MD, USA) and the German Animal Welfare Law (FRG), and approved studies were approved by the animal care and use committees of the BWH Institutional Animal Care and Use Committee. Animals were given free access to food and water with minimal environmental stress and basic environmental enrichment in compliance with our institutional guidelines. The animals were euthanized with intraperitoneal injection of 100 mg/kg ketamine hydrochloride (Sigma-Aldrich, St. Louis, MO, USA) with 10 mg/kg xylazine hydrochloride (Sigma). All experiments were approved by the Regierungspräsidium Karlsruhe Animal Welfare Committee and were conducted in agreement with national and international guidelines.

### Chemical fixation for transmission electron microscopy

Tissues designated for electron microscopy were perfusion fixed for 10 minutes (2% paraformaldehyde and PBS at pH 7.43). The pleura was stained with ruthenium red, lung tissue was submerged in 1% ruthenium red Sigma (Steinheim, Germany) and incubated for 24 hours at 4°C and post-fixed with 1% GA [50]. The post-fixation and ruthenium red labeled samples of the pleura were subjected to reduced OsO4-thiocarbohydrazide-OsO4 (rOTO) method in conjunction with lead aspartate was used for block staining. Briefly, tissue blocks were stained with 1.5% potassium ferrocyanide (Sigma P-8131) and 2% osmium tetroxide (OsO4) in distilled water for 1 h. After washing in distilled water, the samples were incubated in 1% freshly filtered thiocarbohydrazide (Sigma 88535), followed by three rinses with distilled water. Subsequently, the samples were incubated in 2% OsO4 in distilled water for 30 min at 4°C and then washed with water overnight at 4°C. Finally, the sample were washed in 0.1 M cacodylic buffer pH 5.5 for 20 min., incubated with lead aspartate (Merck 11195,) for 30 min at 60°C. The samples were then dehydrated in a in a series of ethanol dilutions (70%, 95% and 100%). Samples were embedded in Epon (Serva, Heidelberg, Germany); 70 nm ultrathin sections were analyzed using a Zeiss EM10 digital transmission electron microscope (Zeiss, Oberkochen, Germany).

### High pressure fixation

Immediately after euthanasia, lung, heart and liver tissue was immersed in DMEM supplemented with 10% FCS and thin slices (200–300 μm) were manually cut using a sharp razor blade. The sections were transferred in an aluminum planchette (Wohlwend, Type A carriers 0.1/0.2 mm) and high pressure frozen (HPM010, Abra Fluid) with FCS supplemented DMEM as a filler.

Freeze substitution of the cells was done using a freeze substitution device EM-AFS2 (Leica Microsystems, Vienna, Austria). The freeze substitution solution used contained 0.1% (w/v) uranyl acetate dissolved in anhydrous acetone and the samples were substituted at -90°C for 72h. The temperature was increased at a rate of 3.5°C/h to -45°C followed by 10h incubation at -45°C. The samples were rinsed with acetone for three times 10 min followed by lowicryl HM20 (Polysciences, Warrington, PA, USA) infiltration with 10%, 25%, 50% 75% lowicryl in acetone for 4h at each step. The temperature was increased to -35°C during the 50% lowicryl step and to -25°C during the 75% lowicryl step. The samples were then left in 100% lowicryl for three times 10h before onset of polymerization. UV polymerization was applied for 24h at -25°C and the temperature was increased to 20°C at a rate of 5°C per hour. The samples were left exposed to UV at room temperature for 24h.

Thin sections (70nm) were cut with a Leica UC6 microtome (Leica Microsystems, Vienna, Austria) and collected on Formvar-coated, copper slot grids. After post-staining in uranyl acetate and lead citrate the sections were imaged using a JEOL JEM-1400 electron microscope (JEOL, Tokyo, Japan) operating at 80 kV and equipped with a 4K TemCam F416 (Tietz Video and Image Processing Systems GmBH, Gautig, Germany).

### Lectin

The biotinylated lectins were obtained from commercial sources. Concavalia ensiformis (ConA) (E1020; lot 10801) [18] was obtained from Dako (Carpinteria, CA, USA). The lectins obtained from Vector Laboratories (Burlingame, CA, USA) were as follows: Lycopersicon esculentum lectin (LEL) (B-1171; lot 80415) [19], Sambucus nigra (SNL) (B-1305; lot 90109) [20], Solanum tuberosum (STA) (B-1161; lot 80330) [21]. Wheat germ agglutinin (GP-2101-10; lot 100610) with 10 nm gold-label were obtained from EY Laboratories (San Mateo, CA, USA).

### Lectin histochemistry

Cryostat sections were obtained from lung specimens perfused with O.C.T. compound and snap frozen. After warming the slide to 27°C, the sections were fixed for 10 minutes (2% paraformaldehyde and PBS at pH 7.43). The slides were washed with buffer (PBS, 5% sheep serum, 0.1% azide, 1mM MgCl_2_, 1mM CaCl_2_) and blocked with 20% sheep serum in PBS. The slides were treated with the lectin followed by avidin-fluorescein ((Southern Biotech, Birmingham, AL, USA) or avidin-fluorescein alone control. The slides were incubated for one hour at 27°C, washed 3 times and mounted with DAPI-containing medium (Vector Labs. Burlingame, CA, USA). The tissue sections were imaged with a Nikon Eclipse TE2000 inverted epifluorescence microscope by using Nikon objectives of 10· and 20· linear magnification with infinity correction. An X-Cite (Exfo, Vanier, Quebec, Canada) 120W metal halide light source and a liquid light guide were used to illuminate the tissue samples.

### Image analysis

The morphometric analysis was performed using Metamorph MetaMorph 7.8 (Molecular Devices, Downington, PA, USA) and ImageJ [51]. Calibrated images were measured using standard MetaMorph tools. Microvillar area density was calculated as the mean microvillar cross-sectional area in serial TEM expressed as a percent surface area of a planar projection of the mesothelium. Similarly, microvillar length was calculated as the mean length measured in serial TEM. The area density of the membrane pits was calculated as the cross-sectional area expressed as a percent surface area of a planar projection of the mesothelium. The skeletonization algorithm utilized the AnalyzeSkeleton (2D/3D) plugin for ImageJ. The ImageJ pruning, path and display functions were optimized based on an MPS test set. Statistical analyses were based on multiple replicates. The unpaired Student’s t-test for samples of unequal variances was used to calculate statistical significance. The data was expressed as mean ± one standard deviation.

### Statistical analysis

The results were compared, processed, and statistically analyzed using MS Excel 365 (Microsoft Corp., Redmond WA, USA) and XLSTAT (Addinsoft, Inc, New York, NY; https://www.xlstat.com/en/). Calibrated morphometric measurements represented normally distributed populations described by the mean and standard deviations of the sample. The data were analyzed suing the Student’s t-test and probability (p) values of <.05 were considered significant.

## Supporting information

S1 Video The video of a high-pressure fixation specimen of murine visceral pleura demonstrates the 3D effect produced by a tilt-angle transmission electron microscope. Note the thickness of the MPS relative to the microvilli. Calibration bar = 1 um.
(AVI)

S2 Video The video of a high-pressure fixation specimen of murine visceral pleura demonstrates the 3D effect produced by tomographic reconstruction of transmission electron microscopy imaging. Note the branched-chain structure of the MPS. Calibration bar = 1 um.
(AVI)

## Abbreviations

ConA: Concavalia ensiformis agglutinin
HPF: high pressure freezing
LEL: Lycopersicon esculentum lectin
MHC: major histocompatibility complex
MPS: mesopolysaccharide
RR: ruthenium red
SNL: Sambucus nigra lectin
STA: Solanum tuberosum agglutinin
TEM: transmission electron microscopy
WGA: wheat germ agglutinin
